# Acute effect of cryoballoon pulmonary vein isolation on the signal-averaged P-wave

**DOI:** 10.3389/fcvm.2025.1728939

**Published:** 2026-01-20

**Authors:** Noah Fantazi, Ben J. M. Hermans, Stef Zeemering, Judith Berbers, Mahdi Emrani, Andras Napp, Nikolaus Marx, Michael Gramlich, Ulrich Schotten, Matthias Daniel Zink

**Affiliations:** 1 Department of Cardiology, University Hospital RWTH Aachen, Aachen, Germany; 2 Department of Cardiology, Cardiovascular Research Institute Maastricht (CARIM), Maastricht University Medical Center (MUMC+), Maastricht, Netherlands; 3 Department of Physiology, Cardiovascular Research Institute Maastricht (CARIM), Maastricht University, Maastricht, Netherlands; 4 Department KPAAIT - Cardiology, Ortenau-Klinikum Offenburg/Lahr, Academic Hospital of Albert-Ludwigs University Freiburg, Freiburg, Germany

**Keywords:** atrial fibrillation, cryoballoon, ECG, pulmonary vein isolation, pulmonary veins, signal-averaged P-wave

## Abstract

**Background and aims:**

Cryoballoon ablation of atrial fibrillation alters a significant amount of vital myocardium contributing to its electrical activity. However, the effect of pulmonary vein isolation (PVI) on the P-wave is unclear. The study aimed to quantify P-wave changes following individual PVI using signal-averaged ECG analysis.

**Materials and methods:**

Unfiltered high-resolution (2,000 samples/second) ECGs were obtained for 5 min pre- and post-isolation of each pulmonary vein in 69 participants using 12 standard and 10 additional leads optimized for atrial electrical activity detection. Signal-averaged ECGs were computed and analyzed using custom software in Matlab. This study was registered at ClinicalTrials.gov (Identifier: NCT06061120).

**Results:**

Significant changes in ECG parameters were found. Following complete PVI, P-wave terminal force measured in lead V1 (3.08 ± 1.99 vs. 2.63 ± 1.76 mV*ms, *p* = 0.003), as well as P-wave complexity (2.05 ± 0.65 vs. 1.79 ± 0.51, *p* < 0.001) and sample entropy (0.210 ± 0.039 vs. 0.187 ± 0.028 au, *p* < 0.001), both averaged across all leads, decreased significantly. Ablation of the left pulmonary veins was associated with lower values in P-wave terminal force (3.07 ± 2.05 vs. 2.84 ± 1.88 mV*ms, *p* = 0.003), P-wave complexity (2.02 ± 0.64 vs. 1.90 ± 0.57, *p* = 0.003), and sample entropy (0.210 ± 0.039 vs. 0.197 ± 0.031 au, *p* < 0.001) after ablation. For ablation of the right pulmonary veins, a decline was observed in sample entropy (0.196 ± 0.031 vs. 0.190 ± 0.027 au, *p* = 0.046).

**Conclusion:**

The observed changes in signal-averaged P-wave parameters during cryoballoon PVI may indicate acute intraprocedural effects on atrial electrophysiology. These exploratory findings suggest that high-resolution, non-invasive ECG recordings are capable to detect stepwise changes of electrical atrial activity, offering new perspectives for intraprocedural assessment of ablation effects.

## 1 Introduction

Atrial fibrillation is the most common sustained heart rhythm disorder in adults (1). Pulmonary vein isolation (PVI) has become an effective therapy since spontaneous focal discharges from the pulmonary veins (PVs) were identified as a major trigger of atrial fibrillation (2). PVI can be achieved by circumferential electrical isolation of the PVs from the left atrium using cryoballoon ablation (3–5). This technique uses low temperatures to create a non-conductive scar around the PVs (4). With this procedure, an acute lesion affecting a substantial portion of the left atrium is formed (6, 7). Therefore, ablation of atrial electrically excitable myocardial mass and isolation of PVs with altered conduction properties (8–18) may result in electrocardiographic P-wave changes (19–24).

However, the acute intraprocedural effects of PVI on atrial electrical activity during sinus rhythm have not yet been systematically characterized. While electroanatomical mapping can provide detailed atrial activation data, it is invasive, costly, and not routinely performed during single-shot procedures like cryoballoon PVI, nor is it feasible for postprocedural follow-up. Standard 12-lead ECGs, although widely available and also tested in this study, lack the temporal and spatial resolution necessary to detect subtle changes in atrial electrical activity (25, 26). Previous studies have assessed P-wave alterations only before and after completion of the entire PVI procedure, without capturing stepwise, intraprocedural changes (19–24).

To address this gap, we used high-resolution, signal-averaged ECGs as a novel, non-invasive and cost-effective method. In contrast to electroanatomical mapping, this technique enables both intra- and postprocedural assessment of atrial electrical activity and may detect subtle changes related to ablation effects (25, 26). The aim of this study was therefore to systematically characterize P-wave changes during sinus rhythm following ablation of each individual PV.

## 2 Materials and methods

### 2.1 Ethics statement

This study was approved by the local ethics committee of RWTH Aachen University (CTC-A-No.: 21-083; Ethics-No.: EK 092/21), registered on ClinicalTrials.gov (ID NCT06061120), and conducted in accordance with the Declaration of Helsinki. Written informed consent was obtained from all subjects. The authors had full access to the data and declare that all supporting data are available in the article. They accept responsibility for the data's integrity and agree to the article as written.

### 2.2 Study design

The trial was a prospective, hypothesis-generating, single-arm, single-center cohort study. Cryoballoon ablations and ECG recordings were performed in 2021 and 2022 at the University Hospital RWTH Aachen. A total of 69 subjects were enrolled in the study. Inclusion criteria were: Documented atrial fibrillation with subsequent planned cryoballoon PVI, ECG recording using YRS-100 device (YourRhythmics, Maastricht, The Netherlands), the ability and willingness to provide informed consent, and an age of at least 18 years. Exclusion criteria were: Previous PVI, non-cardiovertable rhythm other than sinus rhythm during ablation, emergency ablation and unstable condition before ablation.

### 2.3 Study population

**Figure 1:**
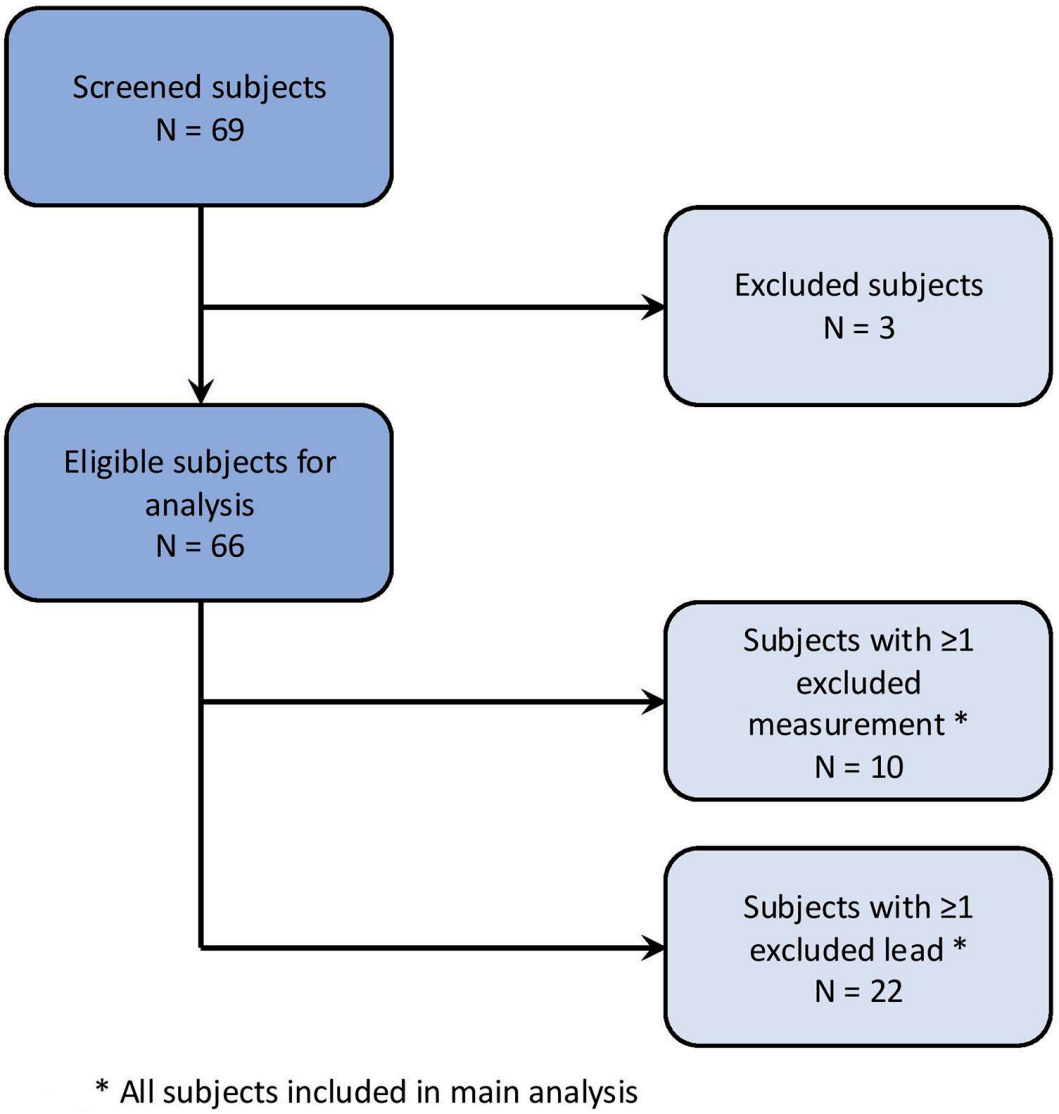
Study population.

The study population is visualized in Figure 1. A total of 69 subjects were initially enrolled. Three subjects were excluded due to a non-cardiovertable rhythm other than sinus rhythm at the begin of the ablation procedure.

Individual ECG recordings were excluded if a non-sinus rhythm was present or if P-wave detection by bespoke software was insufficient due to poor ECG recording quality or external pacing. Individual leads were excluded in cases of insufficient signal quality or electrode disconnection.

Of the remaining 66 subjects, 10 had at least one ECG recording excluded, and 22 subjects had at least one lead excluded from an ECG recording.

### 2.4 ECG recording

**Figure 2:**
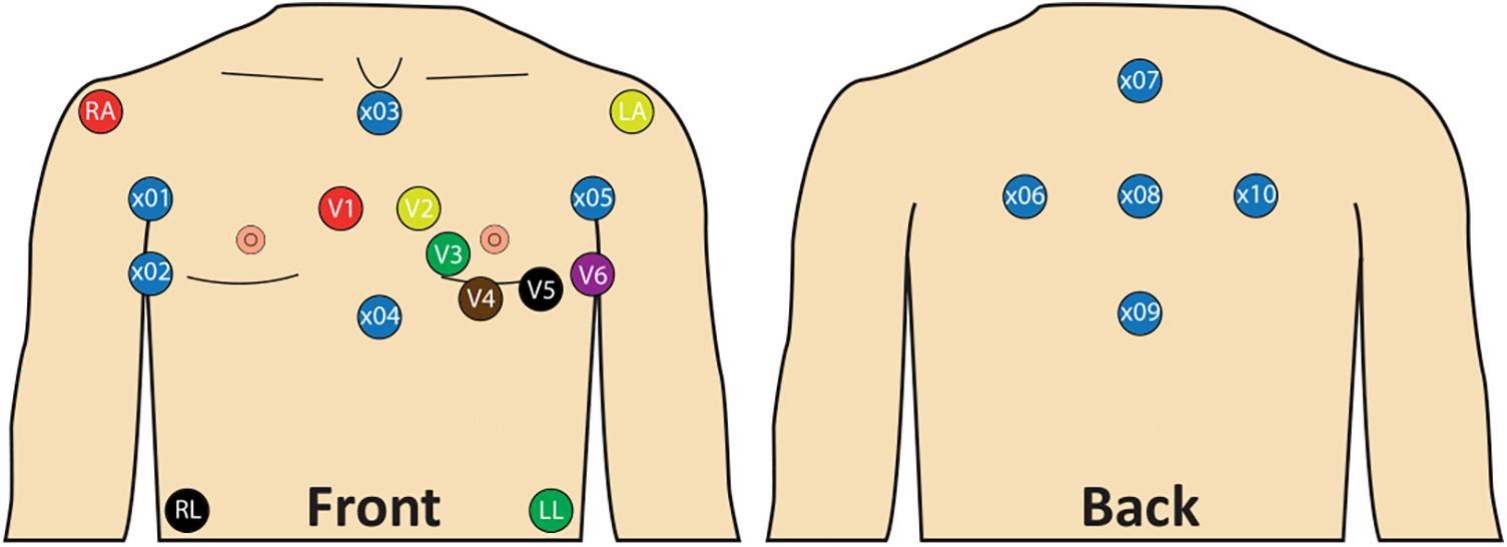
Lead placement with standard 12-lead ECG and 10 additional unipolar leads. x01: Right mid-axillary line 8 cm above x02; x02: Fifth intercostal space, right mid-axillary line; x03: Below jugulum, cranial end of sternum; x04: Xiphoid process; x05: Left mid axillary line 8 cm above V6; x06: Same height as x05, 8 cm left from x08, 4 cm medial to the left posterior axillary line; x07: Vertebra prominens (C7) 8 cm above x08; x08: 8 cm from x06, x07, x09 and x10; x09: 8 cm below x08; x10: Same height as x01, 8 cm right from x08, 4 cm medial to the right posterior axillary line.

ECG recordings were performed with a sampling rate of 2,000 Hz and a duration of at least 5 min per ECG recording. A total of 22 leads were recorded. Of these, 12 leads corresponded to those of a standard 12-lead ECG. Based on prior studies (26–31) 10 additional unipolar leads (×01–10; Figure 2) were included. This lead placement has been shown to maximize derived atrial electrical potential differences (26–31). Landmarks were assigned to the electrode positions to allow for standardization (Figure 2). For the additional lead positions, these included the fifth intercostal space at the right and left mid-axillary line, the manubrium of the sternum, the xiphoid process and the vertebra prominens.

### 2.5 Measurement protocol

**Figure 3:**
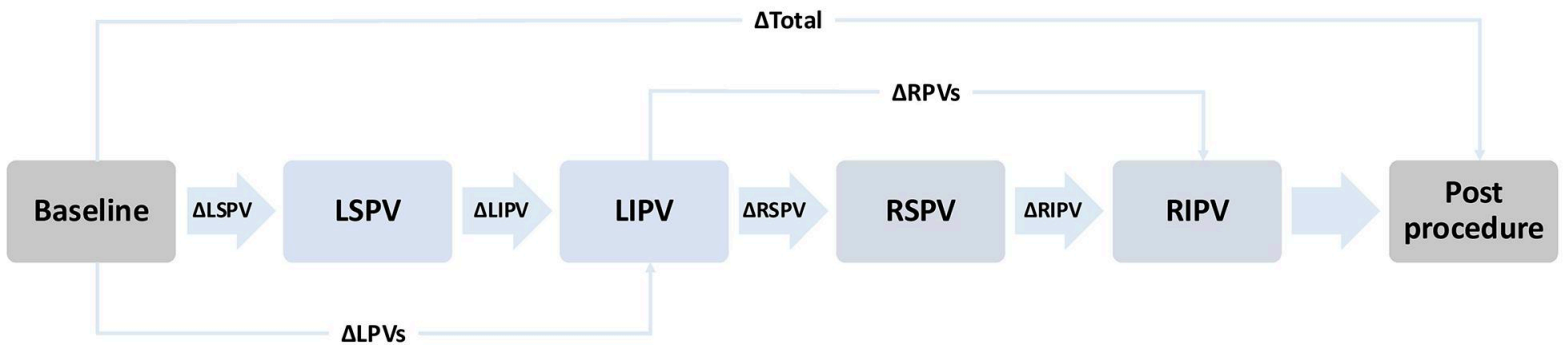
Schematic representation of the measurement protocol. The illustrated order of pulmonary vein isolation (PVI) reflects the most commonly applied order. However, the actual sequence varied depending on the operator. Arrows indicate the order of ablation and the corresponding ECG recordings taken after each pulmonary vein. The Δ symbols represent the statistical comparison performed between the respective measurement timepoints. Baseline: Recording before the start of PVI. LSPV: Recording after ablation of the left superior pulmonary vein. LIPV: Recording after ablation of the left inferior pulmonary vein. RSPV: Recording after ablation of the right superior pulmonary vein. RIPV: Recording after ablation of the right inferior pulmonary vein. Post procedure: Final recording after completion the procedure. LPVs, left pulomary veins; RPVs, right pulmonary veins.

The study protocol was structured as shown in Figure 3. ECG recordings were taken immediately before the start of the procedure (baseline) and after ablation of each PV. The final available recording following the last ablated PV per patient is referred to as the post procedure measurement. The sequence of ablation was at discretion of the treating physician (Supplementary Table S1). Left (LPVs) and right pulmonary veins (RPVs) were always ablated sequentially, typically starting with the LPVs and the left superior pulmonary vein (LSPV). Once the cryoballoon reached a temperature of 20 °C after ablation with subsequent deflation, the ECG recording was started. No manipulation was performed on the subject during the recording. Demonstrated elimination of all PV potentials using a lasso catheter was the endpoint for PVI. The P-waves were compared between baseline and post procedure ECG recordings, between before and after ablation of each PV, and between before and after ablation of LPVs or RPVs. If a left or right common ostium was present, its ablation was included in the comparison of LPVs or RPVs but excluded from the comparison of individual PVs on that side. Subjects with a heart rhythm other than sinus rhythm were electrically cardioverted if possible or excluded from the study.

### 2.6 ECG analysis

#### 2.6.1 Technical background

All ECG recordings were analyzed offline using custom-made software in Matlab (2020b, The MathWorks, Natick, MA, United States). A baseline correction and a 50 Hz notch filter were used as the filter settings. Signal-averaged P-waves were calculated using custom-made algorithms to increase the signal-to-noise ratio (25, 26).

The custom-made algorithm was already described in previous work (26). In brief, a gross alignment of individual beats was performed using the R-peaks. The alignment of P-waves was then finetuned until a maximal correlation between P-waves was achieved. Only P-waves with a correlation of at least 0.9 were considered to automatically exclude premature atrial complexes in the averaged P-wave. A threshold was set to determine the onset and offset of the calculated signal-averaged P-wave. The threshold was defined as the first and last slope ≥150 µV/s. This was done individually for each lead. To calculate the ECG parameters, a global P-wave onset and offset were determined. These were defined as the 10th percentile of the P-wave onset and the 90th percentile of the P-wave offset of all individual leads. QRS-Onset and zero-crossing of the P-wave in V1 were also automatically marked by the customized algorithm. Automatic marking of P-wave onset and offset, zero-crossing, and QRS-onset were confirmed by manual assessment.

#### 2.6.2 ECG parameters

**Figure 4:**
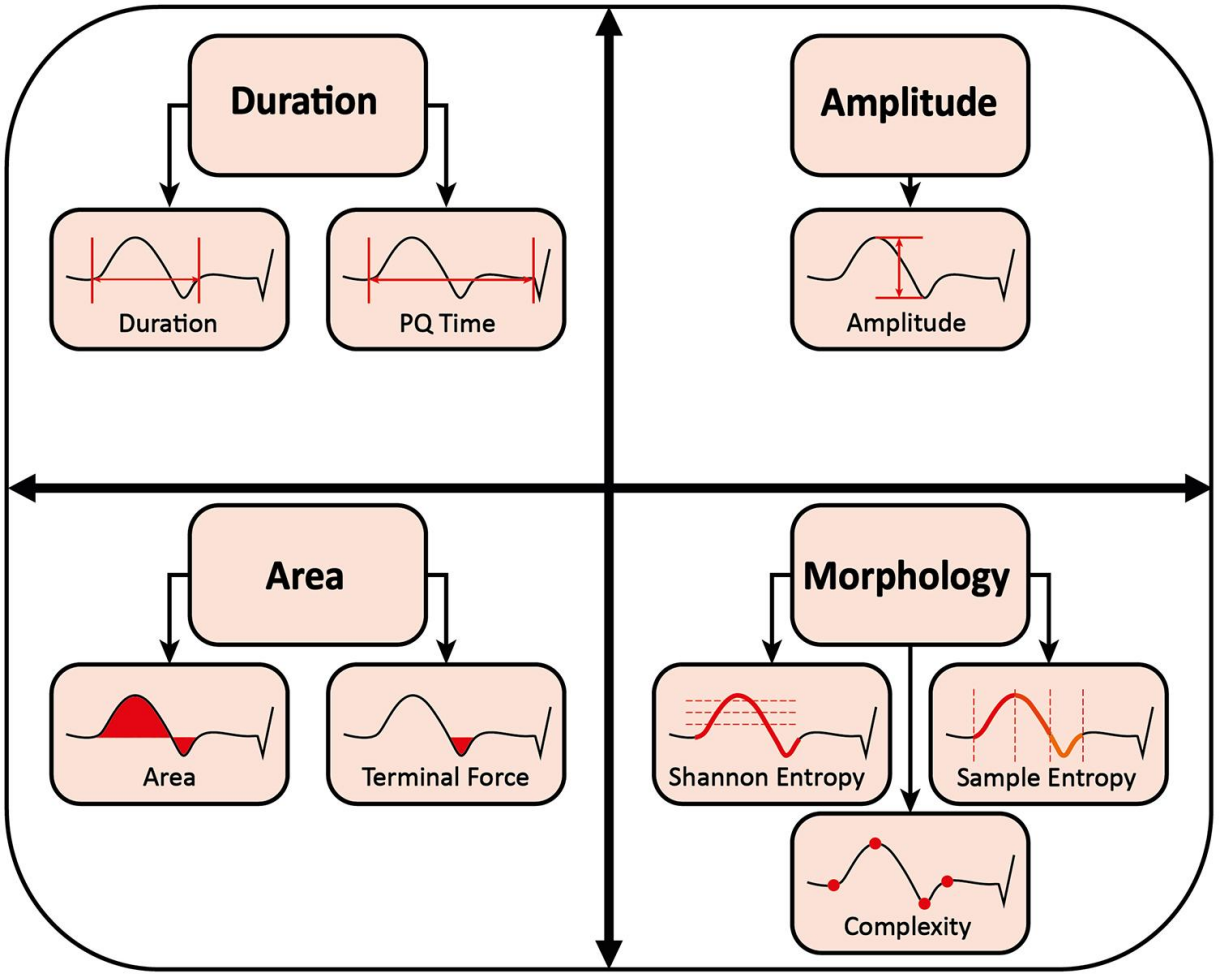
Measurement of P-wave parameters.

In preliminary work, the following ECG parameters were shown to be suitable for describing PVI induced P-wave changes (19–26, 32, 33). The ECG parameters are visualized in Figure 4.

##### 2.6.2.1 P-wave duration and PQ time

P-wave duration was defined as the time from global onset to global end of the signal-averaged P-wave in milliseconds (19–22, 24). The PQ time was defined as the time from the global onset of the calculated P-wave to the QRS-onset in milliseconds (26).

##### 2.6.2.2 P-wave amplitude

P-wave amplitude was defined as the difference between P-wave peak maximum and minimum in millivolts (19).

##### 2.6.2.3 P-wave area and terminal force

P-wave area was defined as the area enclosed by P-wave and isoelectric line in millivolts × milliseconds (19). For the P-wave terminal force, only the negative area between zero-crossing and the end of the P-wave in V1 was considered (19, 23, 24). The P-wave terminal force was shown to represent the electrical activity of the left atrium (32). Previous work has proposed a dV/dt-based delineation of the right-to-left atrial activation transition, validated against electroanatomical mapping (34). For reasons of comparability, we did not adopt this approach and did not introduce any additional temporal segmentation.

##### 2.6.2.4 P-wave morphology

In this study, P-wave complexity, Shannon entropy, and sample entropy are collectively referred to as P-wave morphology (22, 25, 26).

P-wave complexity was defined as the number of significant peaks, negative or positive, within the P-wave (25). Peaks were considered significant if they differed from the neighboring peaks by at least 10% of the average lead-specific P-wave amplitude of all patients (26).

Shannon entropy is the total amount of information in a variable. It quantifies how strongly the data are spread over their possible values in arbitrary units. Thus, it measures the irregularity and uncertainty of a signal. The more spread the data, the greater Shannon entropy and the more irregular and uncertain the signal (26).

Sample entropy is a measure of the regularity and unpredictability of a signal over time-series in arbitrary units. The greater the sample entropy, the more irregular and less predictive the signal (26).

### 2.7 Statistical methods

IBM SPSS Statistics software (IBM Corp. Released 2021. Version 28.0. Armonk, NY, United States) was used for statistical analysis. The Shapiro–Wilk test was used to assess the normality of distribution. For normally distributed continuous variables, a two-tailed paired *t*-test was applied. For non-normally distributed data, the Wilcoxon signed-rank test was used. In Table 2 and throughout the manuscript, a # symbol indicates parameters analyzed with the Wilcoxon signed-rank test. The P-wave terminal force was considered only in lead V1. All other parameters were calculated averaged across all leads. Continuous variables are presented as mean ± standard deviation to ensure consistency and facilitate comparison, even when non-parametric tests were applied. Categorical variables are reported as number and percentage. A *p*-value < 0.05 was considered statistically significant.

## 3 Results

### 3.1 Baseline characteristics

Table 1 summarizes the baseline characteristics of the participants. Most subjects were male (70%) with an average age of 64 ± 10 years. The mean body mass index was 29.1 ± 5.3 kg/m^2^ and the mean CHA2DS2-VA score was 1.9 ± 1.3. While 70% of patients had paroxysmal atrial fibrillation, 79% were in sinus rhythm at the start of the procedure. The average left ventricular ejection fraction was 54 ± 6%, the left atrial diameter was 41 ± 5 mm, and the left atrial area was 22.8 ± 6.3 cm^2^. Most patients were treated with beta-blockers (83%) and oral anticoagulants (94%).

**Table 1:** Baseline characteristics.

Variable	Total
Clinical characteristics	
Male	46 (69.7%)
Age (years)	64.4 ± 10.2
Height (cm)	174.9 ± 10
Weight (kg)	89.6 ± 19.2
Body mass index (kg/m^2^)	29.1 ± 5.3
Paroxysmal atrial fibrillation	46 (69.7%)
SR at start of procedure	52 (78.8%)
CHA_2_DS_2_-VA	1.9 ± 1.3
Congestive heart failure	11 (16.7%)
Hypertension	46 (69.7%)
Age 65–74 years	21 (31.8%)
Age ≥75 years	10 (15.2%)
Diabetes mellitus	8 (12.1%)
Stroke	4 (6.1%)
Vascular disease	16 (24.2%)
Echocardiographic parameters	
LVEF (%)	53.9 ± 5.6
LA diameter (mm)	41.1 ± 4.9
LA size (cm^2^)	22.8 ± 6.3
Antiarrhythmic drug treatment	
Betablocker	55 (83.3%)
Flecainide	6 (9.1%)
Amiodarone	6 (9.1%)
Verapamil	4 (6.1%)
Oral anticoagulation drug treatment	62 (93.9%)

Values are given as mean ± SD or number (percentage). SR, sinus rhythm; LVEF, left ventricular ejection fraction; LA, left atrium.

### 3.2 ECG parameters

**Figure 5:**
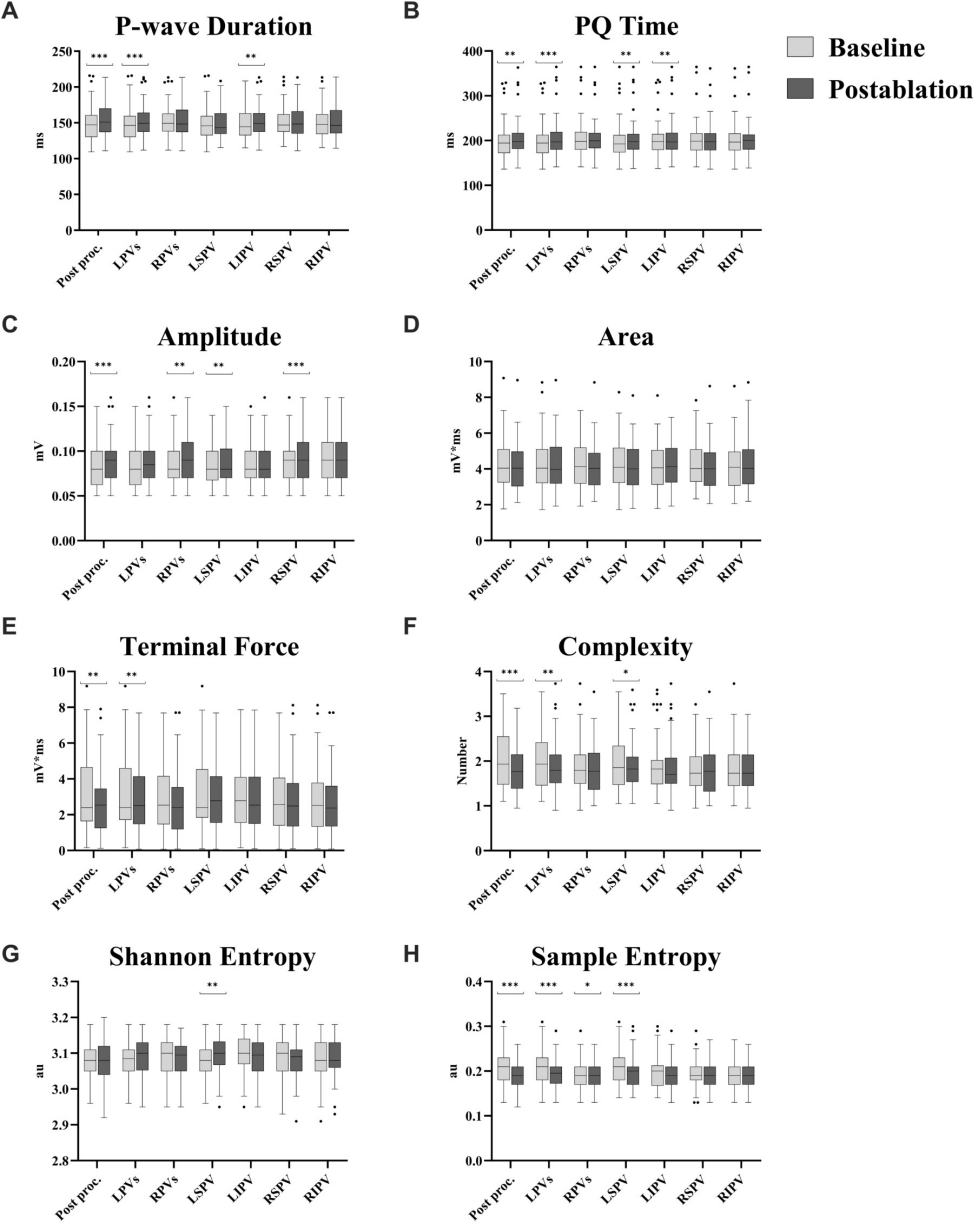
Ablation effect per pulmonary vein for different P-wave parameters. **(A)** P-wave duration. **(B)** PQ time. **(C)** Amplitude. **(D)** Area. **(E)** Terminal force. **(F)** Complexity. **(G)** Shannon entropy. **(H)** Sample entropy. Values are presented as median with interquartile range; whiskers indicate minimum and maximum values, and outliers are displayed individually. All parameters are averaged across all leads, except for P-wave terminal force, which was assessed only in lead V1. Comparison before and after ablation was performed using a two-tailed paired *t*-test or, where appropriate, the Wilcoxon signed-rank test, depending on the result of the Shapiro–Wilk test for normality. Post proc.: Comparison between ECG recordings before (baseline) and after ablation (postablation) of all pulmonary veins. LPVs: Comparison between ECG recordings before the first (baseline) and after the second (postablation) left pulmonary vein ablation. RPVs: Comparison between ECG recordings before the first (baseline) and after the second (postablation) right pulmonary vein ablation. LSPV: Comparison between ECG recordings before (baseline) and after ablation (postablation) of left superior pulmonary vein. LIPV: Comparison between ECG recordings before (baseline) and after ablation (postablation) of left inferior pulmonary vein. RSPV: Comparison between ECG recordings before (baseline) and after ablation (postablation) of right superior pulmonary vein. RIPV: Comparison between ECG recordings before (baseline) and after ablation (postablation) of right inferior pulmonary vein. *: *p* < 0.05. **: *p* ≤ 0.01. ***: *p* ≤ 0.001.

**Figure 6:**
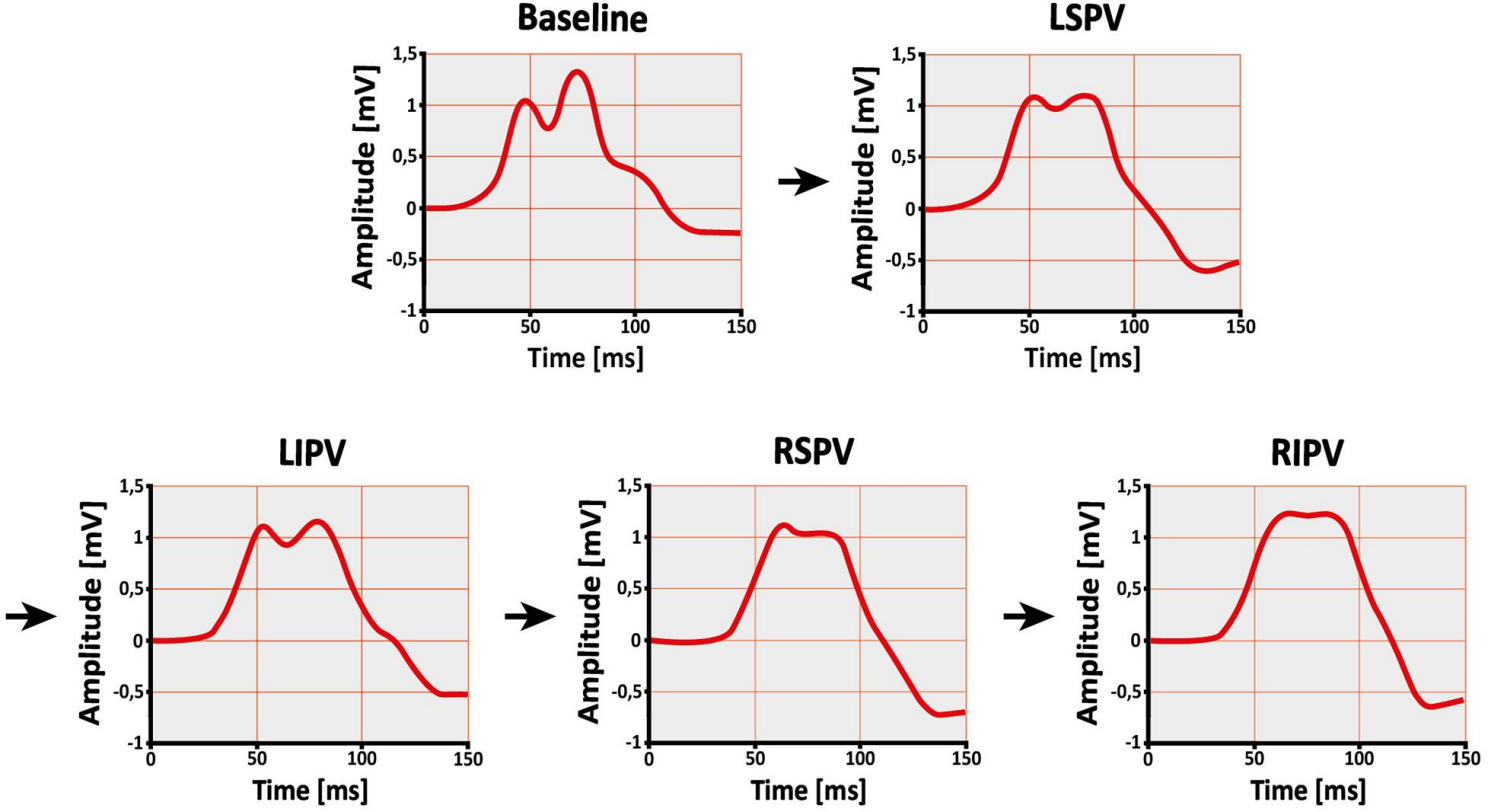
Change in P-wave shape after single pulmonary vein isolation. Example of a subject's signal-averaged P-wave before (baseline) and after ablation of the left superior pulmonary vein (LSPV), left inferior pulmonary vein (LIPV), right superior pulmonary vein (RSPV), and right inferior pulmonary vein (RIPV) in lead II. The order of ablation is indicated by the arrows. As the procedure progresses, the P-wave becomes less complex and irregular.

Changes in all ECG parameters are shown in Table 2 and Figure 5. The signal-averaged P-waves at baseline and after ablation of each PV for a sample subject are visualized in Figure 6.

**Table 2:** Ablation effect per pulmonary vein for different P-wave parameters.

P-wave parameter	Pulmonary vein	Baseline Mean ± SD	Postablation Mean ± SD	*p*
P-wave duration (ms)	Post procedure (*N* = 64)	149 ± 24	154 ± 23	0.001
	LPVs (*N* = 64)	148 ± 24	153 ± 23	<0.001
	RPVs (*N* = 64)	153 ± 22	153 ± 23	0.852#
	LSPV (*N* = 62)	148 ± 23	149 ± 22	0.290
	LIPV (*N* = 62)	149 ± 22	152 ± 22	0.002
	RSPV (*N* = 59)	151 ± 22	152 ± 22	0.353#
	RIPV (*N* = 59)	151 ± 22	150 ± 22	0.438
PQ time (ms)	Post procedure (*N* = 64)	200 ± 41	205 ± 41	0.010
	LPVs (*N* = 64)	200 ± 41	205 ± 43	<0.001#
	RPVs (*N* = 64)	206 ± 43	205 ± 41	0.350
	LSPV (*N* = 62)	201 ± 43	204 ± 43	0.006
	LIPV (*N* = 62)	203 ± 42	205 ± 43	0.008#
	RSPV (*N* = 59)	203 ± 41	202 ± 40	0.347
	RIPV (*N* = 59)	202 ± 42	203 ± 41	0.949#
Amplitude (mV)	Post procedure (*N* = 64)	0.085 ± 0.023	0.091 ± 0.024	<0.001#
	LPVs (*N* = 64)	0.085 ± 0.024	0.088 ± 0.024	0.066
	RPVs (*N* = 64)	0.087 ± 0.024	0.091 ± 0.024	0.002
	LSPV (*N* = 62)	0.085 ± 0.022	0.087 ± 0.024	0.010#
	LIPV (*N* = 62)	0.086 ± 0.023	0.087 ± 0.022	0.820#
	RSPV (*N* = 59)	0.088 ± 0.023	0.092 ± 0.025	<0.001#
	RIPV (*N* = 59)	0.090 ± 0.025	0.092 ± 0.024	0.173
Area (mV*ms)	Post procedure (*N* = 64)	4.28 ± 1.45	4.15 ± 1.34	0.483#
	LPVs (*N* = 64)	4.28 ± 1.50	4.26 ± 1.37	0.832
	RPVs (*N* = 64)	4.25 ± 1.27	4.17 ± 1.31	0.150#
	LSPV (*N* = 62)	4.24 ± 1.40	4.20 ± 1.31	0.875#
	LIPV (*N* = 62)	4.20 ± 1.30	4.22 ± 1.24	0.713#
	RSPV (*N* = 59)	4.28 ± 1.27	4.17 ± 1.30	0.141#
	RIPV (*N* = 59)	4.21 ± 1.32	4.27 ± 1.35	0.277#
Terminal force (mV*ms)	Post procedure (*N* = 63)	3.08 ± 1.99	2.63 ± 1.76	0.003#
	LPVs (*N* = 62)	3.07 ± 2.05	2.84 ± 1.88	0.003#
	RPVs (*N* = 63)	2.82 ± 1.82	2.62 ± 1.73	0.112#
	LSPV (*N* = 61)	3.06 ± 1.95	2.94 ± 1.77	0.056#
	LIPV (*N* = 61)	2.93 ± 1.80	2.82 ± 1.80	0.213#
	RSPV (*N* = 59)	2.81 ± 1.72	2.66 ± 1.81	0.143#
	RIPV (*N* = 58)	2.73 ± 1.87	2.74 ± 1.77	0.388#
Complexity (N)	Post procedure (*N* = 64)	2.05 ± 0.65	1.79 ± 0.51	<0.001#
	LPVs (*N* = 64)	2.02 ± 0.64	1.90 ± 0.57	0.003#
	RPVs (*N* = 64)	1.90 ± 0.58	1.84 ± 0.55	0.639#
	LSPV (*N* = 62)	1.99 ± 0.60	1.89 ± 0.55	0.030#
	LIPV (*N* = 62)	1.92 ± 0.62	1.89 ± 0.59	0.246#
	RSPV (*N* = 59)	1.85 ± 0.55	1.81 ± 0.55	0.463#
	RIPV (*N* = 58)	1.86 ± 0.58	1.83 ± 0.53	0.757#
Shannon entropy (au)	Post procedure (*N* = 64)	3.081 ± 0.050	3.082 ± 0.057	0.839
	LPVs (*N* = 64)	3.083 ± 0.048	3.091 ± 0.052	0.123
	RPVs (*N* = 64)	3.090 ± 0.053	3.083 ± 0.051	0.274
	LSPV (*N* = 62)	3.081 ± 0.049	3.095 ± 0.049	0.006
	LIPV (*N* = 62)	3.096 ± 0.049	3.089 ± 0.053	0.080#
	RSPV (*N* = 59)	3.093 ± 0.054	3.084 ± 0.050	0.242#
	RIPV (*N* = 59)	3.086 ± 0.053	3.088 ± 0.053	0.603
Sample entropy (au)	Post procedure (*N* = 64)	0.210 ± 0.039	0.187 ± 0.028	<0.001#
	LPVs (*N* = 64)	0.210 ± 0.039	0.197 ± 0.031	<0.001#
	RPVs (*N* = 64)	0.196 ± 0.031	0.190 ± 0.027	0.046#
	LSPV (*N* = 62)	0.209 ± 0.037	0.198 ± 0.034	0.001#
	LIPV (*N* = 62)	0.199 ± 0.037	0.196 ± 0.030	0.295#
	RSPV (*N* = 59)	0.194 ± 0.028	0.190 ± 0.027	0.072#
	RIPV (*N* = 59)	0.195 ± 0.031	0.192 ± 0.027	0.192

Values are given as mean ± SD averaged across all derivatives, except for P-wave terminal force, which was assessed only in lead V1. Comparison before and after ablation was performed using a two-tailed paired *t*-test or, where appropriate, the Wilcoxon signed-rank test, depending on the result of the Shapiro–Wilk test for normality. #: Statistical analysis was performed using the Wilcoxon signed-rank test. Post procedure: Comparison between ECG recordings before (baseline) and after ablation (postablation) of all pulmonary veins. LPVs: Comparison between ECG recordings before the first (baseline) and after the second (postablation) left pulmonary vein ablation. RPVs: Comparison between ECG recordings before the first (baseline) and after the second (postablation) right pulmonary vein ablation. LSPV: Comparison between ECG recordings before (baseline) and after ablation (postablation) of left superior pulmonary vein. LIPV: Comparison between ECG recordings before (baseline) and after ablation (postablation) of left inferior pulmonary vein. RSPV: Comparison between ECG recordings before (baseline) and after ablation (postablation) of right superior pulmonary vein. RIPV: Comparison between ECG recordings before (baseline) and after ablation (postablation) of right inferior pulmonary vein.

#### 3.2.1 P-wave duration and PQ time

Both P-wave duration (149 ± 24 vs. 154 ± 23 ms, *p* = 0.001) and PQ time (200 ± 41 vs. 205 ± 41 ms, *p* = 0.010) were prolonged after the procedure. A similar pattern was noted following ablation of the LPVs, with increases in P-wave duration (148 ± 24 vs. 153 ± 23 ms, *p* < 0.001) and PQ time (200 ± 41 vs. 205 ± 43 ms, *p* < 0.001#). In contrast, no differences were detected after RPV ablation for either P-wave duration (153 ± 22 vs. 153 ± 23 ms, *p* = 0.852#) or PQ time (206 ± 43 vs. 205 ± 41 ms, *p* = 0.350). At the level of individual PVs, PQ time was prolonged after both LSPV (201 ± 43 vs. 204 ± 43 ms, *p* = 0.006) and left inferior pulmonary vein (LIPV) ablation (203 ± 42 vs. 205 ± 42 ms, *p* = 0.008#), while P-wave duration increased only after LIPV ablation (149 ± 22 vs. 152 ± 22 ms, *p* = 0.002).

#### 3.2.2 P-wave amplitude

An overall increase in P-wave amplitude was observed during the procedure (0.085 ± 0.023 vs. 0.091 ± 0.024 mV, *p* < 0.001#). This change was primarily linked to RPV ablation (0.087 ± 0.024 vs. 0.091 ± 0.024 mV, *p* = 0.002), whereas the increase following LPV ablation did not reach significance (0.085 ± 0.024 vs. 0.088 ± 0.024 mV, *p* = 0.066). Among single PVs, ablation of the right superior pulmonary vein (RSPV) was associated with an increase in P-wave amplitude (0.088 ± 0.023 vs. 0.092 ± 0.025 mV, *p* < 0.001#), while right inferior pulmonary vein (RIPV) ablation did not result in a significant change (0.090 ± 0.025 vs. 0.092 ± 0.024 mV, *p* = 0.173).

#### 3.2.3 P-wave area and terminal force

No difference in P-wave area was found across the procedure (4.28 ± 1.45 vs. 4.15 ± 1.34 mV*ms, *p* = 0.483#), while P-wave terminal force decreased (3.08 ± 1.99 vs. 2.63 ± 1.76 mV*ms, *p* = 0.003#). Ablation of the LPVs was also associated with a reduction in P-wave terminal force (3.07 ± 2.00 vs. 2.84 ± 1.88 mV*ms, *p* = 0.003#), whereas no change was observed following RPV ablation (2.82 ± 1.82 vs. 2.62 ± 1.73 mV*ms, *p* = 0.112#). In terms of individual PVs, neither LSPV (3.06 ± 1.95 vs. 2.94 ± 1.77 mV*ms, *p* = 0.056#) nor LIPV ablation (2.93 ± 1.80 vs. 2.82 ± 1.80 mV*ms, *p* = 0.213#) showed statistically significant changes.

#### 3.2.4 P-wave morphology

After complete PVI, reductions in both P-wave complexity (2.05 ± 0.65 vs. 1.79 ± 0.51, *p* < 0.001#) and sample entropy (0.210 ± 0.039 vs. 0.187 ± 0.028 au, *p* < 0.001#) were observed. Shannon entropy remained unchanged (3.081 ± 0.050 vs. 3.082 ± 0.057 au, *p* = 0.839).

Ablation of the LPVs was associated with decreases in P-wave complexity (2.02 ± 0.64 vs. 1.90 ± 0.57, *p* = 0.003#) and sample entropy (0.210 ± 0.039 vs. 0.197 ± 0.031 au, *p* < 0.001#). Following RPV ablation, sample entropy also declined (0.196 ± 0.031 vs. 0.190 ± 0.027 au, *p* = 0.046#), while P-wave complexity remained stable (1.90 ± 0.58 vs. 1.84 ± 0.55, *p* = 0.639#).

Only LSPV ablation among the individual PVs showed a reduction in both P-wave complexity (1.99 ± 0.60 vs. 1.89 ± 0.55, *p* = 0.030#) and sample entropy (0.209 ± 0.037 vs. 0.198 ± 0.034 au, *p* = 0.001#).

## 4 Discussion

In our study, we systematically investigated ablation effect on the P-wave using a signal-averaged ECG and additional lead positions during cryoballoon PVI. We observed changes in various ECG parameters during the procedure and explored how ablation of individual PVs was associated with specific alterations in these parameters. Notably, P-wave terminal force tended to decrease after the ablation of LPVs, while P-wave complexity and irregularity were reduced following LSPV ablation. These exploratory findings suggest that non-invasive ECG recordings may reflect ablation effects and could contribute to future evaluation of procedural success, beyond what is currently possible with standard ECG and wearable technologies.

### 4.1 ECG parameters

#### 4.1.1 P-wave duration, PQ time and P-wave amplitude

Both P-wave duration and PQ time were prolonged after LPV ablation, while P-wave amplitude increased particularly after RSPV ablation. In contrast, previous studies have reported a decrease in P-wave duration (19–22, 24) and P-wave amplitude (19) after PVI. Several factors may contribute to these differences.

Different approaches were used to determine the P-wave onset and offset, which may have influenced P-wave duration and PQ time measurements. Additionally, whereas our recordings were obtained immediately after isolation of each individual PV and directly after the final vein, previous studies either did not specify the exact timing of their post-ablation ECG measurements (19, 22) or performed these assessments at later post-procedural time points (20, 21, 24). The earlier timing of our measurements may have captured acute electrophysiological effects that are not assessed by post-procedural or later recordings in previous studies.

One possible factor is myocardial edema formation. Catheter ablation has been shown to cause acute, partially reversible edema in left atrial myocardial tissue (35). Myocardial edema has been associated with transient electrocardiographic changes, potentially affecting conduction properties (36).

Another potential factor is autonomic modulation. The autonomic nervous system exerts a complex influence on cardiac electrophysiology (37). The left atrial ganglionic plexi, which are part of the intrinsic cardiac autonomic nervous system, surround the PVs, among others (38–40). PVI may affect these ganglionic plexi due to their anatomical proximity to common ablation sites (41–44). Beyond direct ganglionic ablation, transient autonomic effects such as vagal reflexes may also play a role (41).

The potentially reversible changes in P-wave duration, PQ time and P-wave amplitude may be due to these circumstances or other unknown mechanisms. As our focus was on actual changes in P-wave parameters rather than their underlying mechanisms, these and the following considerations remain hypothetical.

#### 4.1.2 P-wave area and P-wave terminal force

P-wave area represents total atrial activity, whereas P-wave terminal force is considered specifically as an indicator of left atrial excitation (32). The primary goal of PVI is to electrically disconnect the PVs from the left atrium (3). This procedure results in an absolute reduction of electrically active atrial myocardial mass, as ablative lesions destroy a portion of the atrial myocardium at the PV ostia (6, 7).

Throughout the procedure, P-wave terminal force decreased, while P-wave area remained unchanged. This finding is consistent with previous studies suggesting that the terminal phase of the P-wave is more affected by left atrial electrical mass changes than the total P-wave area (19). The decrease in P-wave terminal force after PVI aligns with earlier research (19, 23, 24).

A decrease in P-wave terminal force was observed after ablation of the LPVs, while no measurable reduction was seen following RPV ablation. Atrial myocardial tissue extends into the PVs, forming sleeves of electrically active myocardium within the PV walls (8). The extent of these myocardial extensions varies, with studies showing that they are more pronounced in the superior and LPVs (13–17). Consequently, isolating these PVs may exclude a larger portion of electrically active myocardium.

Differences in activation timing between RPVs and LPVs may also play a role. The myocardium surrounding the RPVs is activated earlier in sinus rhythm than that of the LPVs (45, 46). Since P-wave terminal force primarily reflects late atrial depolarization (32), electrical activity originating from the RPVs may have a reduced impact on this parameter.

#### 4.1.3 P-wave morphology

The P-wave morphology is much more complex than shown by standard filtered ECGs, which greatly smooth the P-wave (25). As described before, we used the parameters signal complexity (P-wave complexity) and signal irregularity (Shannon entropy and sample entropy) as surrogates for P-wave morphology (26). After the procedure, a reduction was observed in both P-wave complexity and sample entropy, whereas Shannon entropy did not change. This confirms previous observations of changes in P-wave complexity following PVI (22).

Interestingly, these reductions were primarily associated with ablation of the LPVs, particularly the LSPV. As previously discussed, atrial myocardial extensions into the PVs are more pronounced in the superior and LPVs (13–17). In general, myocardial tissue within the PVs is more structurally discontinuous, hypertrophic, and fibrotic compared to the left atrium (15, 16). These properties are associated with heterogenous, slow, and decremental conduction within the PVs (9–12, 18). Removal of such altered conduction patterns from atrial activity by PVI may result in a less complex and irregular P-wave, especially after ablation of LPVs or LSPV. This pattern is consistent with the less pronounced changes observed after ablation of the RPVs described in Section 4.1.2.

### 4.2 Added value and future applications

Currently, procedural success after PVI is typically assessed by monitoring for recurrence of atrial fibrillation. However, this endpoint becomes evident only after a longer observation period and depends on the occurrence of clinical arrhythmia episodes. In contrast, high-resolution signal-averaged ECG may provide insights into ablation-related electrophysiological changes already during sinus rhythm, offering complementary information at an earlier stage and independently of arrhythmia recurrence.

While electroanatomical mapping and cardiac MRI provide superior spatial and mechanistic insights, they are time-consuming, costly, and typically not performed in single-shot ablation procedures. In contrast, high-resolution signal-averaged ECG represents a non-invasive, cost-efficient, and rapidly applicable alternative that may be feasible for repeated peri- and postprocedural use in routine clinical practice.

A key challenge of this study was the electrocardiographic visualization of subtle changes in the left atrium following ablation of individual PVs. To optimize left atrial visualization, additional lead positions were obtained from the front and back based on preliminary studies (26–31). A high-resolution ECG with a sampling rate of 2,000 Hz and a prolonged recording time of 5 min enabled the calculation of a signal-averaged P-wave. While signal averaging substantially improves the signal-to-noise ratio and allows analysis without heavy filtering, it inevitably suppresses beat-to-beat variability and may smooth subtle morphological features. To be independent of the investigator, custom software was used to determine the P-wave onset and offset, as described in the methods section and in previous work (26).

Beyond its potential procedural applications, this concept also relates to the broader framework of atrial cardiomyopathy, which encompasses structural, electrical, and functional remodeling processes predisposing to atrial fibrillation (47). While the present study focused on acute iatrogenic substrate modification during ablation, the same methodological approach could also be applied to study electrophysiological manifestations of chronic pathological remodeling. High-resolution signal-averaged ECGs could therefore represent a promising non-invasive approach to characterize atrial substrate changes over time, whether induced by disease or therapeutic intervention.

To the best of our knowledge, the present study is the first to describe the stepwise electrocardiographic changes in the P-wave during cryoballoon PVI of individual PVs. We found that ablation of the LPVs, particularly the LSPV, was followed by measurable changes in P-wave parameters. Intraprocedural changes in P-wave terminal force and aspects of P-wave morphology were consistent with patterns reported in previous studies analyzing ECG alterations after completed PVI (19, 22–24). While these alterations may be related to short-term electrophysiological remodeling, further research is needed to determine their persistence over time and their potential relevance for evaluating ablation efficacy and predicting long-term rhythm outcomes.

### 4.3 Study limitations

Due to the limited sample size, this study should be regarded as exploratory and hypothesis-generating. Nevertheless, consistent trends in ECG changes were observed, suggesting that larger studies may help confirm and further refine these preliminary findings.

The sequence of PVI was not randomized but followed routine procedural practice, as outlined in Supplementary Table S1. While this reflects common clinical workflows, it may limit the ability to isolate vein-specific effects with certainty. In addition, cumulative effects from preceding ablation steps on later-ablated PVs cannot be excluded. Therefore, the reported changes cannot be conclusively attributed to isolated vein-specific effects.

Intraprocedural ECG recordings may be influenced by transient procedural factors such as edema formation (35, 36) or autonomic responses (37–44). However, these effects are expected to occur in all patients and were mitigated by a standardized protocol: Recordings began only after the cryoballoon reached ≥20 °C and were conducted without catheter manipulation over a continuous 5-minute period. Given the consistent cryoablation technique, observed changes likely reflect a reproducible combination of general procedural effects and localized electrophysiological responses.

Detailed procedural variables such as time-to-isolation or quantitative PV potential characteristics were not consistently available across all veins and were therefore not included. Long-term follow-up was not part of the predefined analysis, as the study focused exclusively on acute intra-procedural surface-ECG changes.

## 5 Conclusion

This study used high-resolution signal-averaged ECGs to evaluate changes in P-wave parameters at multiple defined timepoints during cryoballoon PVI. By acquiring recordings at baseline and after the isolation of each PV, stepwise alterations in atrial electrical activity were observed. These changes, particularly reductions in P-wave terminal force, P-wave complexity, and sample entropy, were most pronounced following ablation of the LPVs and especially the LSPV.

## Data Availability

The raw data supporting the conclusions of this article will be made available by the authors, without undue reservation.
