# Supplementary material for: Acute effect of cryoballoon pulmonary vein isolation on the signal-averaged P-wave
Source: Front Cardiovasc Med. 2026 Jan 20;12:1728939. doi: 10.3389/fcvm.2025.1728939 (PMC12864402; doi:10.3389/fcvm.2025.1728939)
Supplement: Supplementary file 1 [file Table1.docx]

Supplementary Material

**TABLE S1** Distribution of ablation sequences

| Ablation sequence | N |
| --- | --- |
| LSPV 🡪 LIPV 🡪 RSPV 🡪 RIPV | 34 |
| LSPV 🡪 LIPV 🡪 RIPV 🡪 RSPV | 24 |
| LIPV 🡪 LSPV 🡪 RSPV 🡪 RIPV | 4 |
| LIPV 🡪 LSPV 🡪 RIPV 🡪 RSPV | 1 |
| RSPV 🡪 RIPV 🡪 LSPV 🡪 LIPV | 1 |
| RSPV 🡪 RIPV 🡪 LIPV 🡪 LSPV | 1 |
| RIPV 🡪 RSPV 🡪 LSPV 🡪 LIPV | 1 |
| RIPV 🡪 RSPV 🡪 LIPV 🡪 LSPV | 0 |

Distribution of analyzed patients across all observed ablation sequences. Left and right pulmonary veins were always ablated sequentially. In most patients, ablation was initiated at the left superior pulmonary vein. LSPV: Left superior pulmonary vein. LIPV: Left inferior pulmonary vein. RSPV: Right superior pulmonary vein. RIPV: Right inferior pulmonary vein.
